# Vulvitis and Vulvo-Vaginitis in Young Girls

**Published:** 1892-04

**Authors:** 


### VULVITIS AND VULVO-VAGINITIS IN YOUNG GIRLS.

Comby has seen 150 cases of vulvitis and vulvo-vaginitis in girls varying in age from a few months to thirteen years. By far the most frequent cause is contagion, not produced so frequently as is usually stated by attempted coitus, but by the child sleeping with a person suffering from gonorrhœa, or by using the same articles of toilet. The writer states that he has known cases to occur from the child being bathed in the water of a bath previously used by a person having gonorrhœa. Mechanical irritation, such as that caused by masturbation and oxyluria, is rarely the cause, while one of the eruptive fevers, typhoid, eczema or impetigo, may originate the trouble.

Prophylactic measures consist in avoidance of contact with those suffering from gonorrhœa, and, in institutions, thorough cleanliness in regard to urinals, towels, etc. There is both an acute and chronic variety, the former yielding readily to treatment, while the latter requires vigorous measures. The treatment consists in washing the parts two or three times daily with a bichloride solution of the strength of 1 to 2,000, or of acid boracic solution 1 to 25, with the after-application of powdered salol. Sulphur baths are to be used three or four times weekly. In the case of vaginitis, antiseptic crayons, 1-8 inch in diameter, consisting of cocoa-butter, 15 grains, and salol, 1½ grains, are to be introduced into the opening in the hymen every two or three days.—*Rev. Mensuelle des Maladies de L'enfance*, January, 1892—*Annals of Gynœcology.*

